# The Normal Appendix on CT: Does Size Matter?

**DOI:** 10.1371/journal.pone.0096476

**Published:** 2014-05-06

**Authors:** Inneke Willekens, Els Peeters, Michel De Maeseneer, Johan de Mey

**Affiliations:** 1 In vivo Cellular and Molecular Imaging (ICMI) - Vrije Universiteit Brussel, Department of Radiology - UZ Brussel, Brussels, Belgium; 2 Department of Radiology, ASZ Aalst, Aalst, Belgium; 3 Department of Radiology, UZ Brussel, Brussels, Belgium; The Chinese University of Hong Kong, Hong Kong

## Abstract

**Purpose:**

(1) To evaluate the frequency of visualisation and measurements of the normal appendix. (2) To correlate Body Mass Index (BMI) and gender with visualisation of the normal appendix. (3) To correlate age, gender and body length with appendiceal length.

**Materials and Methods:**

A retrospective review of 186 patients undergoing abdominal CT without suspicion of acute appendicitis was done. Frequency of visualisation and measurements (including maximal outer diameter, wall thickness, length, content, location of base and tip) of normal appendices were recorded.

**Results:**

Prevalence of appendectomy was 34.4%. Sensitivity, specificity, positive predictive value, negative predictive value, and accuracy of visualisation of the normal appendix were 76%, 94%, 96%, 67%, and 82% respectively. The mean maximal diameter of the appendix was 8.19 mm±1.6 (SD) (range, 4.2–12.8 mm). The mean length of the appendix was 81.11 mm±28.44 (SD) (range, 7.2–158.8 mm). The mean wall thickness of the appendix was 2.22 mm±0.56 (SD) (range, 1.15–3.85 mm). The most common location of the appendiceal tip was pelvic in 66% appendices. The most common location of the appendiceal base was inferior, medial, and posterior in 37%. The normal appendix contained high-density material in 2.2%. There was a significant correlation between gender and appendiceal length, with men having longer appendices than women.

**Conclusion:**

Most normal appendices are seen at multislice CT using IV contrast. The maximal outer diameter of the normal appendix overlaps with values currently used to diagnose appendicitis on CT.

## Introduction

Acute appendicitis is the most common cause of acute abdominal pain requiring surgery. There is a 6 to 7% lifetime risk to develop appendicitis [1]. A typical clinical presentation occurs only in 50 to 60% of patients [2], [3], [4], [5], [6]. The overall accuracy of clinical diagnosis of acute appendicitis is approximately 80%. The number of unnecessary appendectomies that result from a false-positive clinical diagnosis 13–30%, with a mean false-negative appendectomy rate of about 20% prior to imaging [2], [7], [8], [9], [10], [11], [12], [13], [14], [15], [16], [17], [18], [19], [20], [21], [22], [23], [24], [25], [26], [27], [28]. False-negative appendectomy rates are as high as 15–47% in female patients aged 10–39 years [2], [4], [29].

Imaging can minimize delay in surgical treatment and the subsequent risk of appendiceal perforation [30]. When a normal appendix is visualized on computed tomography (CT) the diagnosis of acute appendicitis is excluded. Hence, it is important to know the frequency of visualization and the appearance of the normal appendix on CT.

Despite the widespread use of CT to diagnose appendicitis, few studies exist that have systematically evaluated the normal appendix [4], [31], [32], [33], [34]. CT criteria for normal size and wall thickness were based on data from the ultrasound literature. A 6-mm short-axis thickness is used as the upper limit of normal. This extrapolation of US findings of a normal appendiceal thickness is based on the size of a compressed and collapsed appendix without taking the luminal content into consideration. CT criteria for luminal content are based in large part on findings on barium contrast studies [4].

The aims of our study were (1) to evaluate the frequency of visualization of the normal appendix, (2) to describe the appearance of the normal appendix (maximal outer diameter, wall thickness, length, intraluminal content, location of the base and tip), (3) to assess whether BMI or gender are related to visualization of the appendix and, (4) to assess whether age, gender, and body length are related to appendiceal length.

## Materials and Methods

### Study population and design

The study was approved by the ethical board. Written informed consent was obtained from all patients. The study was conducted according to the Declaration of Helsinki. Patient records and information was anonymized. A retrospective analysis of abdominal CT scans in 188 consecutive patients undergoing CT of the abdomen was done. There were various indications, however patients with pain in the right lower quadrant or a clinical suspicion of appendicitis were excluded. Our study group consisted of 186 patients (95 men, 91 women; age range, 27–88 years; mean age, 61.64 years ±13.47 [SD]). Of these 188 patients, two were excluded: one because of the presence of metallic artifact from a hip prosthesis, and one because of the presence of motion artifact.

### CT examination and review process

CT scans were obtained with a 16-slice multidetector CT (Sensation 16, Siemens; Erlangen, Germany) with 2 mm collimation and reconstructions every 2 mm. Scanning was performed from the dome of the diaphragm to the pubic symphysis. Iobitridol (Xenetix 350, Guerbet, Roissy, France) or iodixanol (Visipaque 320 GE Healthcare, Amersham, UK) was administered intravenously at a dose of 120 ml and a rate of 2 cc/s. Scans were obtained during the portal venous phase. The protocol was as follows: 120 kVp; 220 mA; sections, 16; section thickness, 2 mm; pitch, 5∶1.5; table speed, 24 mm/sec; gantry speed, 0.5 seconds per rotation.

A radiologist, with more than 5 years of experience in abdominal CT, retrospectively reviewed CT images on a commercially available workstation (Extended Brilliance Workspace; Philips Medical Systems, Best, The Netherlands). The reader was blinded to the patients' surgical history.

Post-processing reformats and measurements were performed using Advance Vessel Analysis (AVA). The coronal and sagittal reformats were reconstructed with sections of 2-mm thickness at 2-mm intervals. The appendix was interpreted as either visualized or non-visualized.

**Figure 1:**
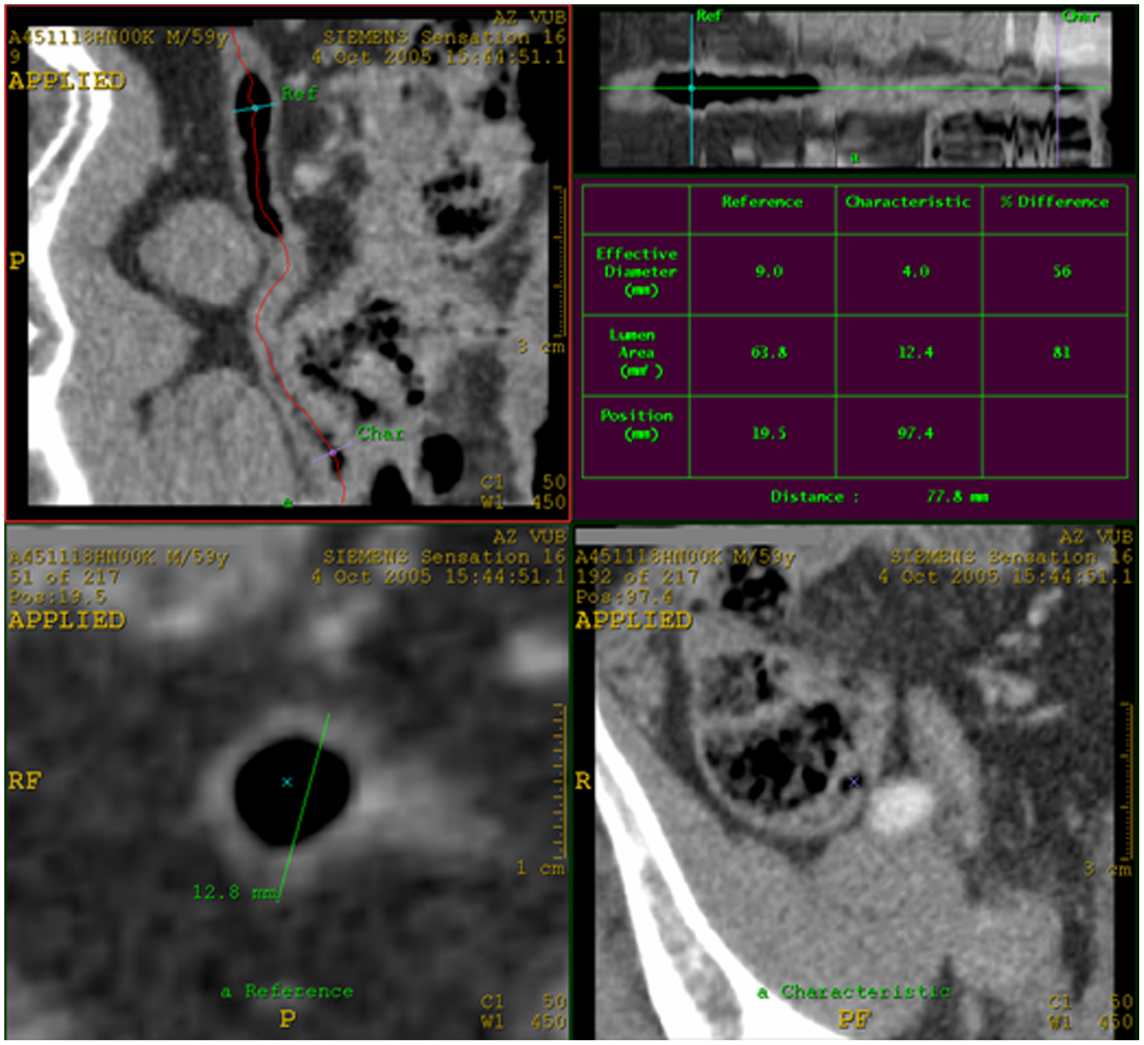
Measurement of maximum outer diameter of the normal appendix on CT. In the top left side viewport the appendix was visualized along its complete length. Here the reader looked for the image truly perpendicular to the axis of the appendix, which corresponded to the maximal outer diameter as seen in the left bottom side viewport. The maximal outer diameter of the appendix was measured in the bottom left side viewport. In the top right side viewport, the appendix was seen as a linear structure.

**Figure 2:**
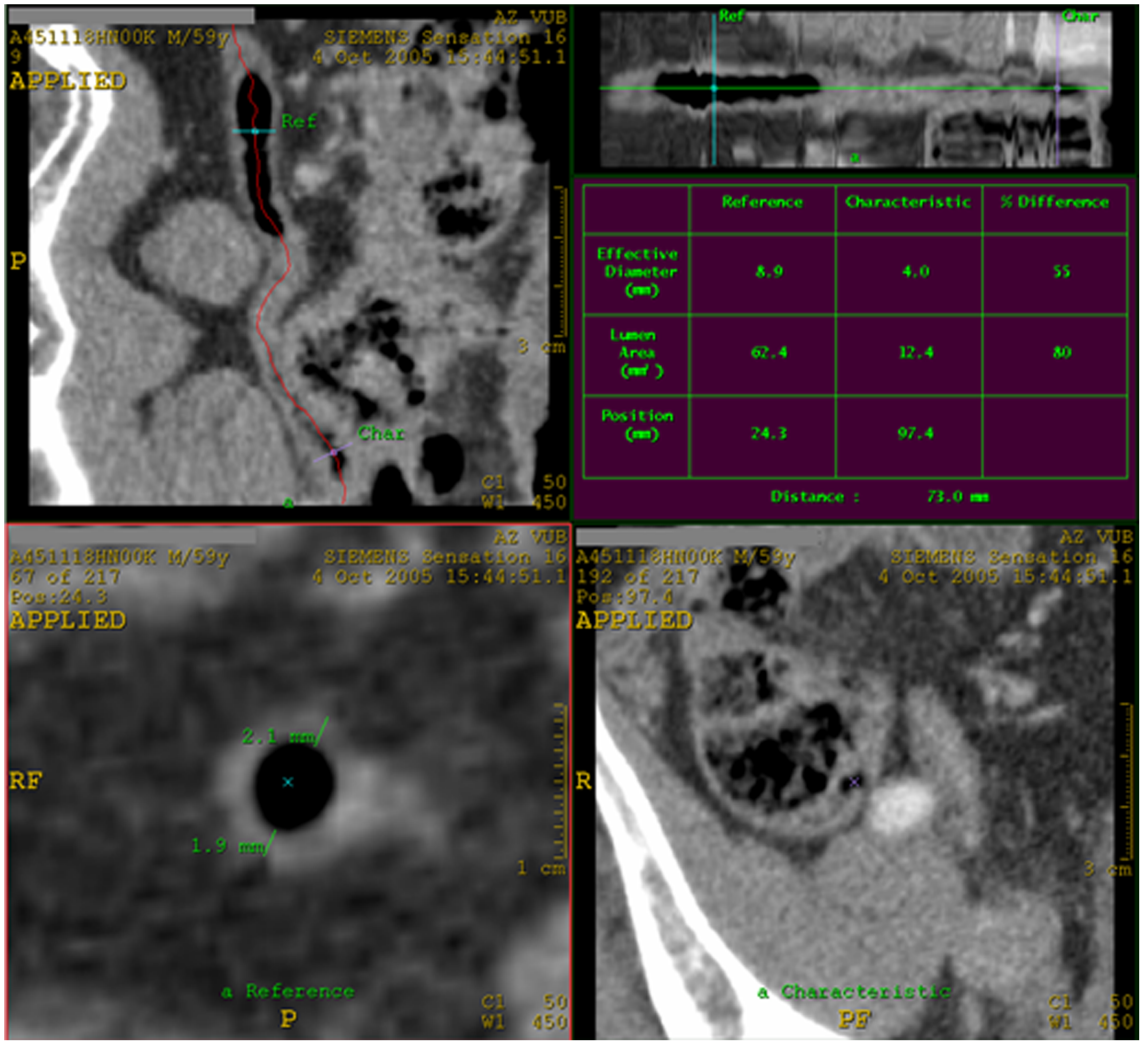
Measurement of the minimum and maximum wall thickness of the normal appendix on CT. In the top left side viewport the appendix was visualized along its complete length. Here the reader looked for the image truly perpendicular to the axis of the appendix, which corresponded to the wall thickness of the appendix. The wall thickness was measured in the two opposite walls on an axial image in the bottom left side viewport. In the top right side viewport, the appendix was seen as a linear structure.

In the Vessel Extraction mode Seed Points were placed in the center of the appendix every other axial slice, thus every other mm. Afterwards, by clicking on Manual Track, the path of the appendix was generated. The appendix was visualized along its complete length and as a curved structure, in the top left side viewport. In the top right side viewport, the appendix was seen as a linear structure. In the top left side viewport, the reader looked for the image truly perpendicular to the axis of the appendix, which corresponded to the largest maximal outer diameter as seen in the left bottom side viewport. The maximal outer diameter of the appendix was measured in the bottom left side viewport (Figure 1). We did not measure the maximal outer diameter in the most proximal and distal part of the appendix. Wall thickness of the appendix was measured in the two opposite walls on an axial image in the same viewport (Figure 2). This was also done in the left bottom side viewport, in a plane truly perpendicular to the axis of the appendix. All measurements were done to the nearest 0.1 mm. In the top right side viewport, the total length of the appendix was measured.

We calculated the mean and range of the average appendiceal wall thickness, of the length of the appendix, and of the maximum appendiceal diameter.

The density of the content of the appendix was measured on axial images. We described it as air, low-density material (<80 HU) or high-density material (>80 HU), or air combined with other material.

The location of the tip of the appendix was described as paracolic, adjacent and along the ascending colon; retrocolic, retrocaecal, behind the colon or caecum; pelvic, extending to the pelvis; midline, or extending to the midline.

The location of the base of the appendix was defined as superior or inferior; anterior or posterior; and medial or lateral with respect to the ileocaecal valve.

Before the examination, every patient was questioned about body length and weight, and history of appendectomy. These data were collected by investigators not involved in the image review process. Body mass index was calculated from the data available in the questionnaire. We also determined mean and range of the body mass index.

### Statistical Analysis

Sensitivity, specificity, negative predictive value, positive predictive value, and accuracy for visualisation of the appendix were determined. The standard of reference for presence of the appendix was obtained by means of clinical history as recorded in the questionnaire.

The Fisher exact test or χ^2^ test and the two-tailed Student T test were used to determine correlation between gender and frequency of appendiceal visualization, as well as to determine correlation between gender and appendiceal length.

Pearson Correlation was used to determine correlation between Body Mass Index (BMI) and frequency of appendiceal visualisation. With the same test we also determined correlation between age and appendiceal length, as well as correlation between body length and appendiceal length.

A significance threshold level of 0.05 was used.

We also evaluated the potential effect of retrocaecal or retrocolic location of the tip of the appendix on the location of the base.

Statistical analysis was performed using commercially available software (IBM SPSS-Statistics).

## Results

The prevalence of appendectomy in this cohort was 34.4% (64 of 186).

Sensitivity for visualization of the appendix was 76%, specificity 94%, negative predictive value was 67%, and positive predictive value 96%. Overall diagnostic accuracy was 82%.

The mean maximal diameter was 8.19 mm±1.6 (SD) (range, 4.2–12.8 mm) (Table 1).

**Table 1:** Diameter, length and thickness of the normal appendix on CT.

	Mean	SD	Minimal	Maximal
Diameter (mm)	8.19	1.6	4.2	12.8
Length (mm)	81.11	28.44	7.2	158.8
Thickness (mm)	2.22	0.56	1.15	3.85

The reviewer was unable to measure the length of the appendix in one patient, because the base could not be identified. The mean length of the normal appendix was 81.11 mm±28.44 (SD) (range, 7.2–158.8 mm) (Table 1). The reviewer was unable to measure appendiceal wall thickness in eight patients, because the density of the lumen was the same as the density of the wall. The mean total thickness of the normal appendix was 2.22 mm±0.56 (SD) (range, 1.15–3.85 mm) (Table 1).

The most common location of the appendiceal tip was pelvic in 62 (66%) of 94 appendices. The appendiceal tip was retrocolic or retrocaecal in 18 (19.5%), paracolic in 8 (8.5%), and midline in 6 (6.4%) (Table 2).

**Table 2:** Location of the appendiceal tip of the normal appendix on CT.

Pelvic	66% (62/94)
Retrocolic/retrocaecal	19.5% (18/94)
Paracolic	8.5% (8/94)
Midline	6.4% (6/94)

The reviewer was unable to localize one appendiceal base. Thus we examined 98.9% (93 of 94) of the cohort. The most common location of the appendiceal base relative to the ileocaecal valve was inferior, posterior, and medial in 34 (37%) of 93 appendices. The appendiceal base was inferior, posterior, and lateral in 16 (17%), inferior and medial in 8 (8.6%), inferior and posterior in 8 (8.6%), inferior and anterior in 4 (4.3%), inferior and lateral in 4 (4.3%), inferior, anterior, and lateral in 4 (4.3%), inferior, anterior, and medial in 3 (3.2%), inferior, and posterior in 2 (2.2%), and superior, anterior, and medial in 1 (1%) of patients (Table 3).

**Table 3:** Location of the appendiceal base of the normal appendix on CT.

Inferior	Superior	Inf/Sup	Medial	Lateral	Med/Lat	Anterior	Posterior	Ant/Post
94.6% (88/93)	2.2% (2/93)	2.2% (2/93)	58.1% (54/93)	26.9% (25/93)	15.1% (14/93)	18.3% (17/93)	68.8% (64/93)	12.9% (12/93)

We compared the location of the appendiceal base of the retrocaecal and retrocolic tips with the location of the bases of the other tips (pelvic, paracolic and, midline). The bases of the retrocaecal tips were located inferior in 87.5% (7 of 8) and superior in 12.5% (1 of 8); medial in 62.5% (5 of 8), lateral in 12.5% (1 of 8) and, midline in 25% (2 of 8); anterior in 0% (0 of 8), posterior in 62.5% (5 of 8) and midline in 37.5% (3 of 8). The bases of the retrocolic tips were located inferior in 81.2% (9 of 11), superior in 9% (1 of 11) and midline in 9% (1 of 11); medial in 54.5% (6 of 11) and lateral in 45.4% (5 of 11); anterior in 18.2% (2 of 11), posterior in 72.7% (8 of 11) and midline in 9% (1 of 11). The locations of the bases of the other appendiceal tips were inferior in 97.3% (72 of 74), superior in 1.4% (1 of 74) and 1.4% midline (1 of 74); medial in 59.5% (44 of 74), lateral in 25.7% (19 of 74) and midline in 14.9% (11 of 74); anterior in 20.3% (15 of 74), posterior in 68.9% (51 of 74) and midline in 10.8% (8 of 74).

The locations of the appendiceal bases of the retrocaecal/retrocolic tips were mostly inferior, medial and posterior which is similar to the locations of the bases of the other (pelvic, paracolic, and midline) tips.

The normal appendices contained air and low-density material in 44.7% (42 of 94), low-density material in 22.3% (21 of 94), were completely air-filled in 17% (16 of 94), contained air and high-density material in 13.8% (13 of 94) and, high-density material in 2.2% (2 of 94) (Table 4).

**Table 4:** The content of the normal appendix on CT.

Air + Low density	44%(42/94)
Low density	22% (21/94)
Air	17% (16/94)
Air + High density	13% ((13/94)
High density	2% (2/94)

The mean BMI of our study population was 23.76±3.29 (SD) (range, 17.2–36.89), consistent with mildly overweight patients making up our study group. There was no statistically significant correlation between BMI and visualisation of the normal appendix (P value 0.264), between gender and visualisation of the normal appendix (P value 0.218), between age and length of the normal appendix (P value 0.188), between gender and length of the normal appendix (P value 0.13), and between body length and length of the normal appendix (P value 0.281).

The mean appendiceal length was 88.32 mm±29.81 (SD) in men and 72.42 mm±26.98 (SD) in women.

## Discussion

In this study, sensitivity for visualisation of the normal appendix on CT with IV contrast administration was 76%; specificity was 94%; positive predictive value 96%; negative predictive value67%; and accuracy 82%.

In previous studies using CT, the appendix could be identified in 36% to 94% of individuals [4], [14], [32], [34], [35], [36], [37], [38], [39], [40], [5], [10], [18], [33], [41].

The highest sensitivity (>90%) for visualisation of the normal appendix is obtained when rectal or oral contrast is administered [33], [36], [41]. Yet, rectal contrast requires catheterization. This procedure may be uncomfortable for patients, and time consuming for radiology technicians. Failure of rectal contrast to reach the caecum also has been reported in as many as 18% [30] of individuals. Rectal contrast may also have a risk of appendiceal perforation [41]. Rectal contrast administration is contraindicated in neutropenic patients,those with peritoneal signs, and evidence of perforation [7].With fluoroscopic barium enemas up to 20% of normal appendices do not fill. [42]. Oral contrast often is tolerated poorly and may delay treatment by several hours [43], [44], as it takes 45 minutes to 2 hours for the contrast material to reach the caecum [40], [41], [45]. Oral contrast is poorly tolerated by patients with nausea, resulting in further delay.

An important advantage of IV contrast is that it allows a complete assessment of other abdominal pathologic conditions [46]. Rhea found that other conditions were diagnosed in 34% to 80% of patients [47].

In this study, the mean maximal diameter was 8.19 mm±1.6 (SD) (range, 4.2–12.8 mm). Other studies have shown that the mean diameter of a normal appendix is 5.6+/−1.3 mm to 6.6+/−1 mm [4], [10], [33]. Although patients in our study group may have had appendicitis that spontaneously resolved this appears unlikely given that a high percentage of appendices (86 (91.5%) of 94) were greater than 6 mm. None of the patients in our cohort had a diagnosis of appendicitis at discharge. Our findings suggest that in the absence of other CT signs of appendicitis, a diameter of 6 mm is not a reliable cut off value. A normal appendix with air or contrast material distension is reported to have a transverse diameter of up to 11 mm [33], [34], [38]. In this study, more than 42% of patients had an appendiceal diameter greater than 6 mm [4].

Other authors have suggested a threshold of 10 mm [33], [34], [38], in particular when luminal contents are not visualised, and in the absence of periappendiceal inflammation [34], [38]. Our results are in accordance with the findings of these authors. We measured appendiceal diameter truly perpendicular to the axis of the appendix. Our method of measurement has not been employed in previous studies. In our study the normal appendix had a mean full thickness of 2.22 mm±0.56 (SD) (range, 1.15–3.85 mm). The maximum mural thickness of the appendix has been reported as less than 2–3 mm [51]. Wall thickening beyond 3 mm may be considered a sign of inflammation [4]. Previous reports showed that 0.9% of normal appendices had a wall thickness of 3 mm or greater [4]. In our study a wall thickness of more than 3 mm was found in 8% (7 of 86).

Our findings show that the mean length of the normal appendix is 81.11 mm±28.44 (SD) (range, 7.2–158.8 mm). According to reports in the surgical literature the appendiceal length can vary from 20 to 200 mm, averaging 6–10 cm [48], [49], [50]. We showed no correlation between appendiceal length and age or body length. It has been suggested that the appendix is longer in children and may become smaller after mid-adult life.

We found a correlation between appendiceal length and gender. Longer appendices were observed in men. To our knowledge no previous studies have examined appendiceal length on CT.

The location of the appendix is variable. The most common location of the appendiceal tip was pelvic in 62 (66%),retrocolic in 11 (12%), paracolic in 8 (8.5%), retrocaecal in 7 (7.5%), and midline in 6 (6.4%). In an anatomopathological study of 10000 subjects, the vermiform appendix was retrocaecal and retrocolic in 65.28%, pelvic in 31.01%, subcaecal in 2.26%, preileal in 1%, and postileal in 0.4% [52]. Our findings are not in accordance with the belief that the most common location of the appendix is retrocaecal [52].

The most common location of the appendiceal base was inferior, medial, and posterior in 34 (37%) of 93 appendices. The bases of the retrocaecal and retrocolic tips were most commonly located inferior, and medial. This location is the same as that of the other (pelvic, paracolic, and midline) tips. Normal development and migration of the appendix from an anterior position during fœtal life and childhood to a more posterior location during adulthood explains this finding. Migration of the base is related to faster growth of the anterior and lateral walls of the caecum [52].

Most normal appendices have recognizable intraluminal content. Only 17% were completely air-filled, and 58.5% were partially air-filled. However, the presence of air does not exclude appendicitis [4]. Intraluminal air is a common finding in both the normal and inflamed appendix. 2.2% of appendices contained high-density material and 13.8% (13 of 94) contained air and high-density material. No appendicoliths were found.-Although appendicoliths show a significant association with appendicitis, this finding is not specific. Appendicoliths are found in 1.7% of patients with a normal appendix. 86.7% (13 of 15) of patients with high-density material in the lumen underwent a previous study with contrast. In two patients these data were unavailable.

Our study has some limitations. A main limitation is the absence of pathologic correlation, and the use of patient history as the gold standard. Patients may have had their appendix removed previously and not remember this information, although this is rather unlikely.

Some of these patients may have congenital absence of the appendix. However, given the low incidence (1/100000) of agenesis this appears unlikely in our study group.

In conclusion, our study showed that most normal appendices are seen on multislice CT after IV administration of contrast agent. The normal diameter of the appendix can be as high as 12.8 mm. 91.5% of normal appendices are larger than 6 mm in our study. The normal wall thickness is larger than 3 mm in 8% of normal appendixes.

Hence, relying on appendix size alone may lead to misdiagnosis and mismanagement.
